# The diagnostic value of the pulsatility curve to predict shunt responsiveness in patients with idiopathic normal pressure hydrocephalus

**DOI:** 10.1007/s00701-022-05233-7

**Published:** 2022-05-30

**Authors:** M. W. T. van Bilsen, L. van den Abbeele, V. Volovici, H. D. Boogaarts, R.H.M.A. Bartels, E. J. van Lindert

**Affiliations:** 1 Department of Neurosurgery, Radboud University Medical Center, Geert Grooteplein-zuid 10, 6525GA, Nijmegen, The Netherlands; 2 Department of Neurosurgery, Erasmus Medical Center, Rotterdam, The Netherlands

**Keywords:** Normal pressure hydrocephalus, Pulsatility curve, Lumbar infusion testing

## Abstract

**Objective:**

The aim of this study was to investigate the diagnostic accuracy of the pulsatility curve to predict shunt response in patients with idiopathic normal pressure hydrocephalus (iNPH).

**Methods:**

Lumbar cerebrospinal fluid dynamics were derived from an automatic lumbar infusion test (LIT) protocol. All patients were treated with ventriculoperitoneal shunting and re-examined 6 months after shunting. Patient demographics and outcomes were gathered in a prospective, electronic database that spanned from January 2012 to January 2020. A validated iNPH scale was used to assess patients preoperatively and 6 months postoperatively. The relationship of the relative pulse pressure coefficient (RPPC), delta amplitude, successful lowering of amplitude, and the pressure-value at a hypothetical amplitude of zero (P_0_), resistance to outflow (R_out_), and outcome, were assessed using receiver operating characteristic (ROC) curves.

**Results:**

We included 38 patients. The RPPC, delta amplitude, successful lowering of amplitude, and P_0_ parameters did not predict shunt response. Mean P_0_ was 0.5 (IQR 0.4–0.9) in improved patients and 0.4 (IQR 0–1.2) in non-improved patients. The delta amplitude was 0.16 kPa (IQR 0.10–0.23) in improved patients and 0.18 kPa (IQR 0.11–0.24) in non-improved patients. Furthermore, we found a technical failure rate of pulsatility curve measurements of 32%.

**Conclusion:**

Pulsatility curve results were not suitable in predicting shunt response in our cohort. The diagnostic value of LIT in case of normal pressure hydrocephalus should be subject to more rigorous research.

**Supplementary Information:**

The online version contains supplementary material available at 10.1007/s00701-022-05233-7.

## Introduction

Lumbar infusion testing (LIT) is a diagnostic test that is currently used to predict positive shunt response in patients suspected of idiopathic normal pressure hydrocephalus (iNPH). However, current LIT derivates are unable to predict negative shunt response [2, 5, 8, 11, 12, 18, 20, 22] and, therefore, LIT might not be suitable to exclude patients from shunting.

Previous literature [16] suggested that better predictive accuracy could be achieved by looking at the pulsatility curve. The pulsatility curve is the linear relationship between different CSF-pressure values and the corresponding signal amplitudes, both after fluid drainage and during the infusion. It has been suggested that pulsatility curves could offer a new way of thinking in the pathophysiology of iNPH [10] and its derivates as delta amplitude and the pressure-value at a hypothetical amplitude of zero (P_0_) could possibly increase sensitivity and specificity in predicting shunt response [15].

In reaction to these data, CELDA®, who is the manufacturer of the Likvor CELDA® automated lumbar infusion testing System (CELDA, Likvor AB, Umeå, Sweden), altered its protocol in 2015 with the adding of pulsatility curve measurements. However, no data about the diagnostic value of this alteration exists in current literature.

This is, to our knowledge, the first study that explores the diagnostic accuracy of the pulsatility curve to predict shunt response in patients with iNPH. A prospective, electronic database that included patients from January 2015 to January 2020 was used.

## Methods

The study was approved by the Medical Ethics Commission of the Radboud University Medical Center (no. 2020–6615).

### Hypothesis

The hypothesis of this study was that a high delta amplitude would be a good diagnostic marker for a positive shunt response, and a low amplitude or inability to lower the amplitude would correlate with a negative shunt response. We also hypothesized that combining delta amplitude with other diagnostic markers deduced from LIT as P_0_, Rout and tap test, could improve specificity and sensitivity.

### Pulsatility curve

**Fig. 1:**
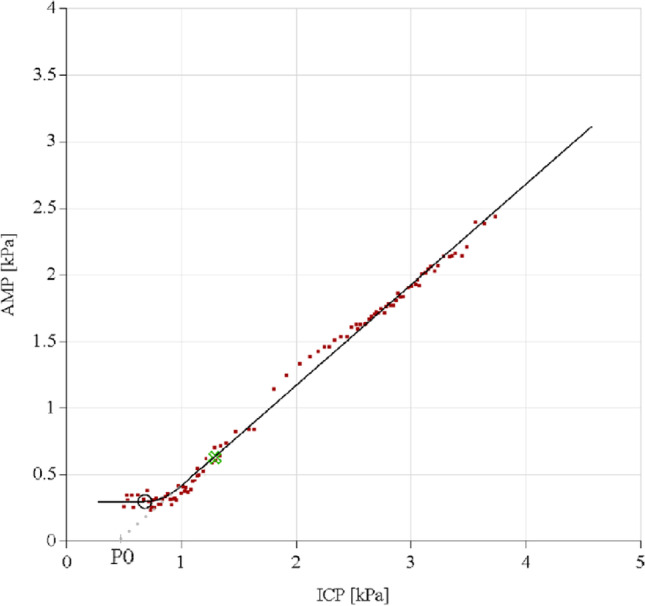
Example of a pulsatility curve with constant phase. The green cross indicates resting pressure and amplitude. The black circle indicates pressure and amplitude after CSF retraction

**Fig. 2:**
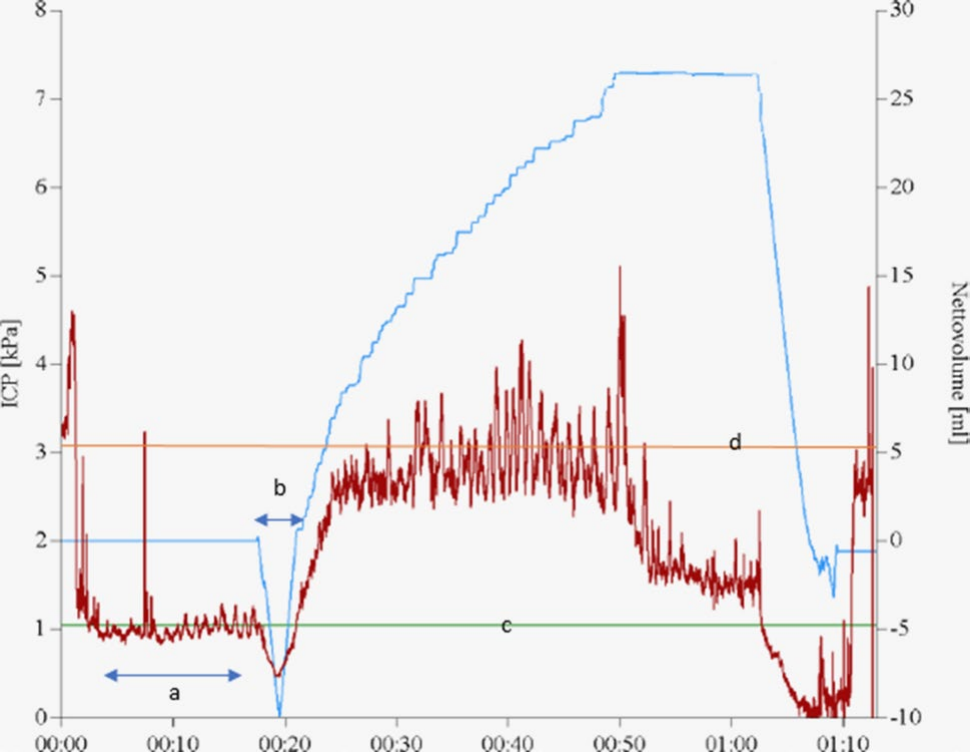
Example of a standard LIT measurement reading. a. 00:05–00:10 Calculation of baseline pressure (kPa); b. 0:19–0:21 fluid withdrawal for pulsatility curve and delta amplitude calculations; c = baseline pressure; d = plateau pressure; Rout = (d-c)/infusion rate

**Fig. 3:**
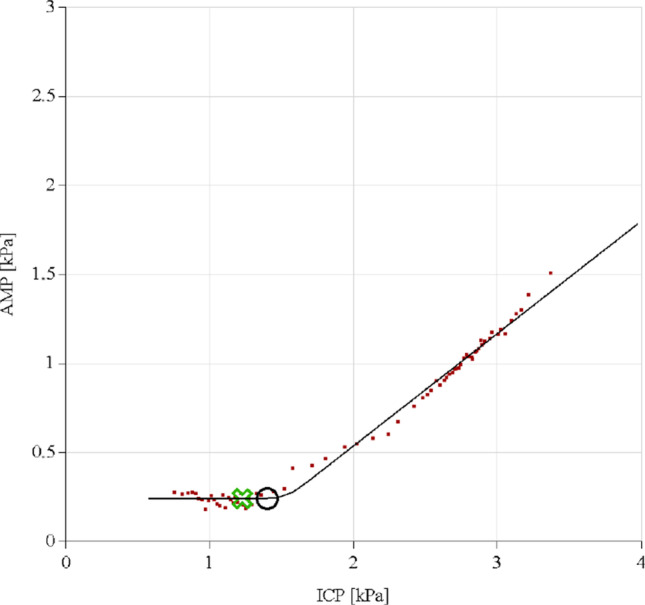
Example of a pulsatility curve with constant phase, in which the green cross indicates resting pressure and amplitude. The black circle indicates pressure and amplitude after CSF retraction. There is an inability to achieve any lowering of amplitude when lowering CSF-pressure

*Delta amplitude* was defined as the difference between the baseline pulse amplitude (bAmp (kPa)) and constant phase amplitude (cAmp (kPa)) of the pulsatility curve (Fig. 1). The pulsatility curve plots the linear relationship between CSF-pressure (*x*-axis) and signal amplitude of cardiac-related pulsations in CSF-pressure (*y*-axis). However, lower pressure levels, which are reached after the withdrawal of CSF, give a minimum level of pulse amplitude and further decline in CSF-pressure that will not yield lower amplitude levels [16]. This results in a constant phase after lowering pressure by withdrawal of CSF (Figs. 1 and 2). In practice, some patients fail to achieve any lowering of their amplitude when lowering CSF-pressure (Fig. 3). This could explain their failure to respond to shunting, and therefore, in theory, a low or absent delta amplitude could predict a negative shunt response.

*P*_*0*_ is described as the pressure value when the hypothetical amplitude of zero would be reached (Fig. 1). Comparisons in earlier literature between healthy subjects and iNPH patients yielded no difference in pulsatility curve slope. Nonetheless, previous literature did describe a significant shift to the left of the curve, resulting in a significantly lower P_0_ in iNPH patients compared to healthy subjects [10]. Therefore, shunt responders could have a lower P_0_ compared to non-responders.

Both delta amplitude and P_0_ of the pulsatility curve could, thus, be a predictor of negative shunt response [15]. In reaction to this literature, the Likvor CELDA® System (CELDA, Likvor AB, Umeå, Sweden) altered its protocol in 2015 with the addition of pulsatility curve measurements and withdrawal of CSF for 2 min before start of infusion. The protocol aimed at a total reduction to 0.8 kPa below resting CSF-pressure to reach a constant phase [21].

### Study design

The Radboud University Medical Center, Nijmegen, the Netherlands, is a tertiary academic center with a catchment area of 1.7 million people. One of the areas of special interest at the center is hydrocephalus care. Referred patients with the suspicion of normal pressure hydrocephalus undergo a standardized evaluation by a specialized physician assistant under the supervision of the senior author (EL). The results of this evaluation are prospectively collected in a digital database and include sex, age, comorbidity, MRI, date of LIT, LIT outcomes as R_out_, and baseline pressure, 50 cc tap test outcomes, date of shunt implantation, pre- and postoperative symptoms, gait evaluation, mRS, mmse and moca, type of shunt, subjective improvement after shunting, and surgical complications. Post-operative evaluations were performed routinely six to 8 weeks after surgery and 6 months after surgery. Symptoms were scored according to the NPH-score of Kubo et al. [13] (Supplementary Table 1). Improvement after shunting was defined as at least one point improvement in NPH-score.

Lumbar infusion tests were performed with the use of the automated infusion protocol of the Likvor CELDA® System (Umeå, Sweden) (Fig. 1).

Patients who underwent shunt surgery preceded by a LIT according to the new pulsatility curve protocol, i.e., between January 2015 and July 2020 for the diagnosis of idiopathic normal pressure hydrocephalus (iNPH), were included in this study. Patients with secondary NPH or aqueductal stenosis were excluded.

**Fig. 4:**
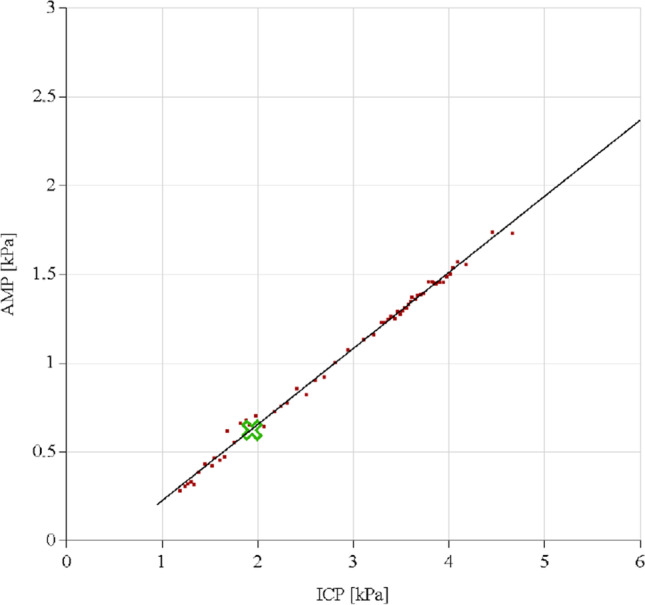
Example of a pulsatility curve without constant phase, in which the green cross indicates resting pressure and amplitude. There is an ability to achieve any lowering of amplitude when lowering CSF-pressure, but no constant phase was reached

Pulsatility curves were retrospectively analyzed by the first author (MB), who was during this analysis blinded to the results of the shunting procedure. Data were analyzed following the example illustrated in Figs. 1, 3, and 4 for baseline pressure (bP), infused volume, resistance to outflow (Rout (mmHg/ml/min)), baseline pulse amplitude (bAmp(kPa)), amplitude in the constant phase after fluid retraction (cAmp)(kPa)), total retracted fluid (ml), pressure after fluid retraction (aP), delta amplitude (bAmp-cAmp(kPa)), and slope of the linear phase of the curve (RPPC). If a constant phase was not reached, a linear decrease in delta amplitude after fluid retraction was also considered a possible positive predictor of shunt response (Fig. 4), according to the Celda Guidance in reading pulsatility curve results [21].

### Shunt implantation

Evaluation of clinical symptoms and data of the lumbar infusion test and tap test were used to inform patients about their chance of positive shunt response. No indications for shunt surgery were made based on the pulsatility curve or delta amplitude. Patients who chose to undergo shunt implantation received a ventriculoperitoneal shunt with a Miethke GAV 5/30 valve (Aesculap AG, Tuttlingen, Germany).

### Statistical analysis

For statistical analyses, R (version 3.6.1, The R project for statistical computing, Vienna) was used.

Because the analysis of the study data was retrospective and the cohort was relatively small, we performed a post hoc power analysis using Gpower 3.1. We calculated the necessary sample using an expected mean difference of 0.25 (and 0.25 standard deviation per group) in delta amplitude between the two groups (responders and non-responders). This difference is relatively large, as is the standard deviation, which was 0.11 and 0.13 in the two groups, respectively. A number of 22 patients yielded 90% power to detect 0.25 difference in delta amplitude; hence, we consider our study sufficiently powered.

Descriptive analyses were run for all variables. Continuous variables were tested for normality using the Shapiro–Wilk test. Means and standard distributions (SDs) were reported for continuous variables and medians and interquartile ranges (IQRs) were reported for non-normally distributed variables. A point-biserial correlation was also performed for the subjective outcome variable and the continuous postoperative NPH score. The variable with the best correlation to the subjective improvement variable was chosen as the primary outcome, with the other one serving as the sensitivity analysis. Improved and non-improved patient characteristics were compared using the Wilcoxon rank sum test for non-normally distributed variables and the *t*-test for normally distributed variables. The “epiR” package was used to calculate the diagnostic accuracy measures for different cut-offs of the delta amplitude. We built generalized linear models by regressing the delta amplitude, Rout, preoperative NPH, P0, and RPPC on the dichotomized outcome. A receiver operating characteristic (ROC) curve was then built, and the area under the curve (AUC) calculated. We also built calibration curves using the “givitR” package.

## Results

A total of 38 patients could be included in the study after the introduction of the new protocol with pulsatility curve measurements in 2015 (Table 1). Post hoc power analysis was performed (supplementary Fig. 1), which showed an adequate sample size. Median patient age was 72 (interquartile range (IQR) [68, 76]). Median pre-operative NPH-score was 5.0 (IQR 5.0, 7.0). All patients had walking difficulties, 32 patients (84%) had cognitive problems, and 31 (82%) patients had micturition problems.

**Table 1:** Characteristics in patients with shunting for iNPH

Characteristic	All patients (N = 38)	Improved (N = 26)	Non-improved (N = 12)
Age (median [IQR])	72.00 (68.00, 76.00)	73.00 (69.25, 77.00)	70.00 (65.75, 74.25)
Sex (F/M)	14/24	8/18	6/6
Comorbidity (%)			
Vascular	14 (36.8)	11 (42.3)	3 (25.0)
Diabetes	8 (21.1)	5 (19.2)	3 (25.0)
Hypertension	11 (28.9)	9 (34.6)	2 (26.7)
Pre-existing neurological disease or stroke	12 (31.6)	6 (23.1)	6 (50.0)
Time between LIT and shunting in months (median [IQR])		1.00 (1.00, 2.00)	1.50 (1.00, 3.50)
Preoperative NPH score (median [IQR])	5.00 (5.00, 7.00)	5.50 (5.00, 7.00)	5.00 (4.75, 5.25)
Preoperative walking difficulties (%)			
No walking difficulties	0 (0)	0 (0)	0 (0)
Dysbasia or drift, no objective disturbance	3 (7.9)	3 (11.5)	0 (0)8 (66.7)
Unstable, but independent gait	14 (36.8)	6 (23.1)	3 (25.0)
Walking with support	19 (50.0)	16 (61.5)	
Walking not possible	2 (5.3)	1 (3.8)	
Preoperative cognitive problems (%)			
Normal cognitive ability	6 (15.8)	3 (11.5)	0 (0)
Subjective amnesia or inattention	18 (47.4)	6 (23.1)	8 (66.7)
Objective amnesia or inattention	11 (28.9)	16 (61.5)	3 (25.0)
Time/place disorientation	3(7.9)	1 (3.8)	1 (8.3)
Meaningful conversation impossible	0 (0)	0 (0)	0 (0)
Preoperative micturation problems (%)			
Normal micturition	7 (18.4)		1 (8.3)
Pollakiuria or urgency	11 (28.9)		5 (
Incontinence 1–3 times a week	9 (23.7)		2
Daily urinary incontinence	8 (21.1)		3
Completely deficient	3 (7.9)		1
Improved after tap test		20 (76.9)	9 (75.0)
mmse before tap test (median [IQR])	26.00 (24.00, 27.25)	26.00 (24.00, 28.00)	27.00 (24.50, 27.00)
moca before tap test (median [IQR])	21.00 (20.00, 24.00)	21.00 (20.00, 23.00)	24.00 (18.50, 26.00)
mmse after tap test (median [IQR])	27.00 (26.00, 28.00)	27.50 (25.25, 28.00)	27.00 (27.00, 29.00)
moca after tap test (median [IQR])	23.50 (21.00, 26.00)	22.00 (21.00, 25.00)	26.00 (24.00, 27.00)
Preoperative mRS (%)			
0	2 (5.9)	1 (4.2)	1 (10)
1	19 (55.9)	13 (54.2)	6 (60.0)
2	10 (29.4)	8 (33.3)	2 (20.0)
3	1 (2.9)	1 (4.2)	0 (0.0)
4	2 (5.9)	1 (4.2)	1 (10.0)
5	0 (0)	0 (0.0)	0 (0.0)

In 18 of the 38 patients who underwent the protocol with pulsatility curve measurement, a constant phase could be reached. In eight patients, a successful lowering without constant phase was observed. In the remaining 12 patients, system errors led to unsuccessful measurements, resulting in a technical failure rate of 32%.

All patients received a ventriculoperitoneal shunt with a Miethke GAV 5/30 valve (Aesculap AG, Tuttlingen, Germany).

Median follow-up duration was 6 months (IQR 6–8). Improvement after shunting was defined as at least one point improvement of NPH-score. No significant differences between improved patients and non-improved patients were found concerning the different LIT-derivates.

Median P_0_ was 0.5 (IQR 0.40–0.85) in improved patients and 0.35 (IQR − 0.03–1.25) in non-improved patients (*p*-value 0.653) and could be considered as similar. Median RPPC was 0.60 (0.44–0.65) in improved patients and 0.58 (IQR 0.45–0.66) in non-improved patients (*p*-value 1.0). The delta amplitude was 0.16 kPa (IQR 0.10–0.23) in improved patients and 0.18 kPa (IQR 0.11–0.24) in non-improved patients (*p*-value 0.915). Resting pressure was 1.40 kPa (IQR 1.12–1.80) in improved patients and 1.35 kPa (IQR 1.08–1.63) in non-improved patients (*p*-value 0.670), with a corresponding rest amplitude of 0.46 kPa (IQR 0.29–0.66) in improved patients and 0.42 (IQR 0.28–0.54) in non-improved patients (*p*-value 0.520). The Rout was 13.10 mmHg/ml/min (IQR 10.85–19.20) in improved patients and 11.45 mmHg/ml/min (IQR 10.25–13.18) in non-improved patients (*p*-value 0.432).

Six adverse events (16%) were registered after a shunting procedure. Three patients underwent revision of the peritoneal catheter due to abdominal shunt dislocation. One patient had an intraparenchymal hemorrhage seen on standard postoperative CT, without complaints. One patient suffered from a subdural hematoma, postoperatively. One patient underwent valve replacement due to valve dysfunction.

## Discussion

This study is, to our knowledge, the first diagnostic study of a new LIT protocol with pulsatility curve measurement in relationship to the effect of a shunting procedure. It was hypothesized that pulsatility curve could predict shunt responsiveness in patients with iNPH.

### Summary of results

The results of the study did not support the study hypothesis. Correlations between delta amplitude, successful lowering, or P_0_, and shunt response were not statistically significant.

### CSF-hydrodynamics

One year after Hakim first described the syndrome of NPH in 1964 [6], he postulated together with Adams [7] the idea of altered hydrodynamics as a potential pathophysiological mechanism in NPH.

Soon after Hakim’s discovery, it became clear that not all patients suspected of normal pressure hydrocephalus responded to a shunting procedure and shunting entailed serious intraoperative and postoperative adverse events. In 1978, Hughes et al. [9] described an improvement rate of 33% and an adverse event rate of 35%. Therefore, in order to prevent shunting complications in patients who would not benefit from shunting, the hypothesis of changed hydrodynamics contributed to the development of lumbar infusion testing as a diagnostic tool to differentiate shunt responders from non-responders in suspected NPH patients [4, 19].

Up to now, different LIT derivates as R_out_, plateau pressure, and amplitude have shown a various, but overall somewhat disappointing ability to predict negative shunt response in suspected iNPH [8, 14, 22]. The suggestion that the pulsatility curve is a promising LIT derivate to predict shunt response is relatively new [10, 15–17].

In 2010, Anile et al. [1] did find a difference in RPPC between responders and non-responders. They pointed out that the slope of the curve had a sharp cut-off of 0.25 between improved and unimproved patients. However, these results could not be reaffirmed in 2018 by Nabbanja et al. [14]. Also, in the study of Delwel et al. [3], they did not show any relation between RPPC and effect of shunting. However, these results could be due to methodological differences, such as small sample size, study inclusion differences, and differences in methods used.

In order to investigate the concept further, Jacobsson et al. studied the differences in LIT values between patients suspected of iNPH and healthy controls in 2018 [10]. They found no difference in RPPC or amplitudes, but observed a shift of the pressure-amplitude curve to the left in patients suspected of iNPH, as amplitudes were higher for same ICP compared to healthy controls. This led to the hypothesis that patients with iNPH indeed had deviant pulsatility curve, but no difference in slopes. In order to define differences between iNPH patients and healthy controls, they advocated a different analyzation of pulsatility curves. They suggested that patients who responded to shunting would have higher amplitudes at a hypothetical pressure of zero (P_0_) and successful reduction of amplitude with reduction of ICP by CSF withdrawal, which they called delta amplitude. These pulsatility curve measurements were implemented in the Celda lumbar infusion system protocol [21], which was used in our center.

Therefore, in our study, we aimed initially to investigate the hypotheses of pulsatility curve measurements to predict shunt response in iNPH patients in clinical practice.

We unfortunately could not confirm predictive value of pulsatility curve measurement, and therefore, our results did not support the study hypothesis. If indications were led by the pulsatility curve derivates, patients would have been unfairly denied surgery.

### Limitations

Confidence intervals were large, and the numbers used for the analysis were small. However, suspected differences were not found and we do not suspect that a bigger sample size would result in the high sensitivity and specificity needed to lead surgical decision. Another big drawback of the pulsatility curve measurements in our practice was the high technical failure rate of 32%. This was in concordance with the high chance of technical failure indicated in the Celda pulsatility curve manual [21], which stated that measurement of the pulsatility curve is highly sensitive to needle problems when retracting CSF, resulting in system errors and inability to measure delta amplitudes. Possibly, this is due to patient factors, such as lumbar stenosis, in which pressure warnings can be triggered by rootlets touching the sensor when CSF is drained. Operator errors can also not be excluded as influencing factors, as no comparison is available in current literature and all measurements in our population were performed by the same investigator (LA). Unfortunately, other than the Celda manual, no comparable data in the literature was found. Jacobssen et al. [10] did perform CSF extraction, as they did show a constant phase, but they did not show delta amplitude data of failure rates. To overcome technical problems in our patients, sensors were always connected to the best draining needle and LIT was performed by a trained and specialized physician assistant (LA). However, considering these fallouts, the technical failure rate is a drawback in pulsatility curve as a diagnostic tool for iNPH. Therefore, protocol improvements are required for further studies on predictive accuracy of pulsatility curves, especially regarding delta amplitude.

LIT was originally designed with the goal of excluding patients from shunting, in order to minimize risks associated with shunting in this fragile patient population. However, its use may be counterproductive nowadays, as risks of shunting are decreased and shunting leads to improvement in over 70% of patients in current literature with minimal lasting, serious adverse events. This is supported by our study, in which complication rate was relatively low and effect of shunting was high with no differences in success between patients who underwent LIT and those who did not undergo LIT.

### Conclusion

Pulsatility curve results were not suitable to predict shunt response in our cohort. The diagnostic value of LIT in case of normal pressure hydrocephalus should be subject to more rigorous research.

## Supplementary information

Below is the link to the electronic supplementary material.

Supplementary file1 (DOCX 13 KB)

Supplementary file2 (TIFF 195 KB)

Supplementary file3 (TIF 71 KB)

## Abbreviations

iNPH: Idiopathic normal pressure hydrocephalus
NPH: Normal pressure hydrocephalus
CSF: Cerebrospinal fluid
LIT: Lumbar infusion testing
P_0_: Pressure-value at a hypothetical amplitude of zero
R_out_: Resistance to outflow
bAmp: Baseline pulse amplitude (kPa)
cAmp: Constant phase amplitude (kPa)
mRS: Modified Rankin Scale
mmse: Mini-mental state examination
moca: Montreal cognitive assessment
RPPC: Relative pulse pressure coefficient
ROC: Receiver operating characteristic
SDs: Standard distributions
IQRs: Interquartile ranges
AUC: Area under the curve
