# Supplementary material for: The diagnostic value of the pulsatility curve to predict shunt responsiveness in patients with idiopathic normal pressure hydrocephalus
Source: Acta Neurochir (Wien). 2022 May 30;164(7):1747–54. doi: 10.1007/s00701-022-05233-7 (PMC9233651; doi:10.1007/s00701-022-05233-7)
Supplement: Supplementary file 1 — Supplementary file1 (DOCX 13 KB) [file 701_2022_5233_MOESM1_ESM.docx]

| **Table 1. NPH-score** | | | |
| --- | --- | --- | --- |
|  | **Cognition** | **Walking** | **Micturition** |
| **0** | Normal | Normal | Normal |
| **1** | complaints of amnesia or inattention but no objective memory and attentional impairment | complaints of dizziness of drift and dysbasia, but no objective gait disturbance | pollakisuria or urinary urgency |
| **2** | existence of amnesia or inattention but no disorientation of time and place | unstable, but independent gait | occasional urinary incontinence (1-3 or more times per week but less than once per day |
| **3** | existence of disorientation of time and place, but conversation is possible | walking with any support | continuous urinary incontinence (1 or more times per day) |
| **4** | disorientation for the situation or meaningful conversiation impossible | walking not possible | bladder function is almost or completely deficient |
