# Supplementary figures and images for: The diagnostic value of the pulsatility curve to predict shunt responsiveness in patients with idiopathic normal pressure hydrocephalus
Source: Acta Neurochir (Wien). 2022 May 30;164(7):1747–54. doi: 10.1007/s00701-022-05233-7 (PMC9233651; doi:10.1007/s00701-022-05233-7)

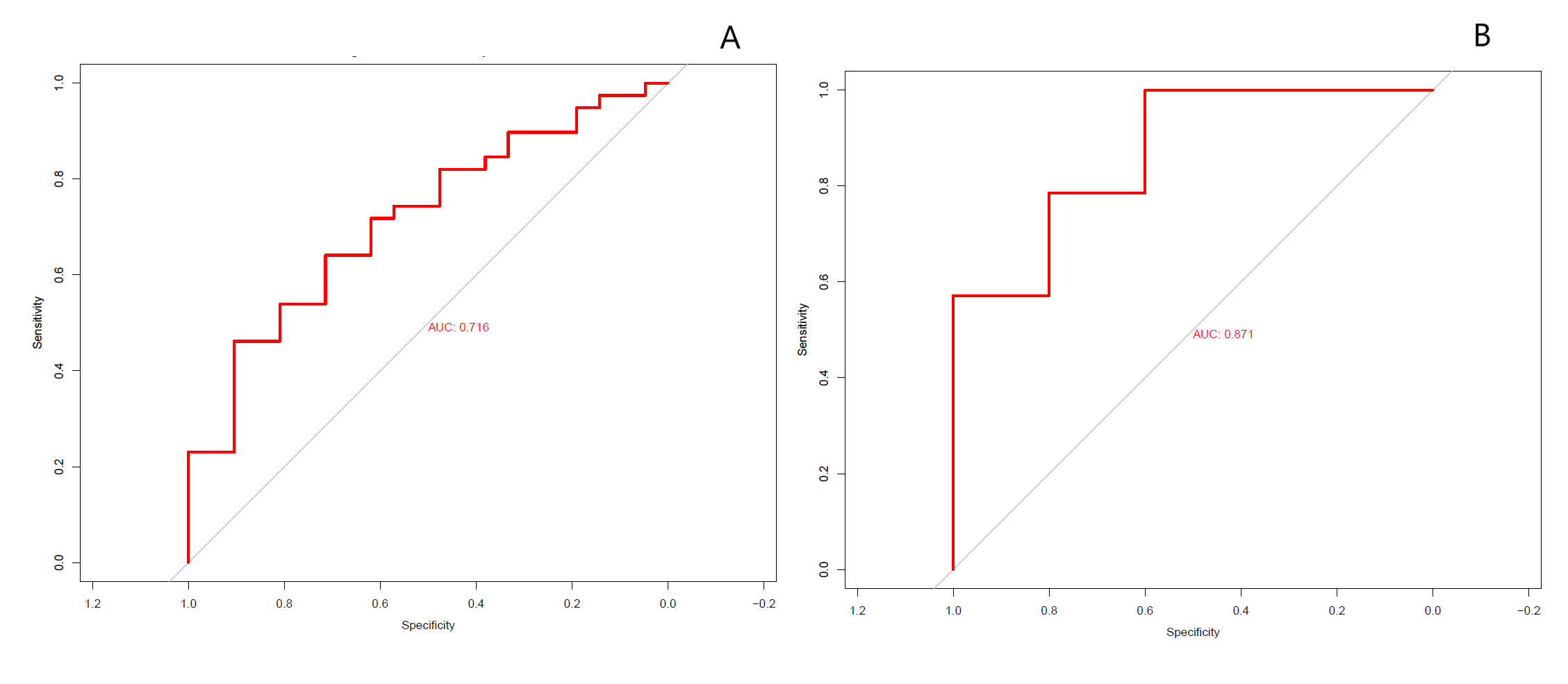

Supplement: Supplementary file 2 — Supplementary file2 (TIFF 195 KB) [file 701_2022_5233_MOESM2_ESM.tiff]

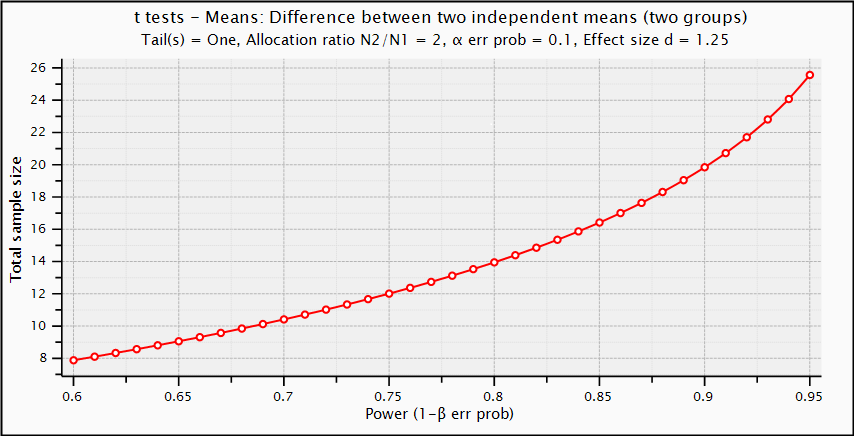

Supplement: Supplementary file 3 — Supplementary file3 (TIF 71 KB) [file 701_2022_5233_MOESM3_ESM.tif]
